# New insight into the role of macrophages in ovarian function and ovarian aging

**DOI:** 10.3389/fendo.2023.1282658

**Published:** 2023-11-06

**Authors:** Maoxing Tang, Manzhi Zhao, Yuhua Shi

**Affiliations:** ^1^ Department of Reproductive Medicine, Guangdong Provincial People’s Hospital (Guangdong Academy of Medical Sciences), Southern Medical University, Guangzhou, China; ^2^ Department of Pulmonary and Critical Care Medicine, Guangdong Provincial People’s Hospital (Guangdong Academy of Medical Sciences), Southern Medical University, Guangzhou, China

**Keywords:** macrophages, ovarian aging, ovarian function, follicle development, inflammation, female infertility

## Abstract

Macrophages (MΦs) are the most abundant leukocytes in mammalian ovaries that have heterogeneity and plasticity. A body of evidence has indicated that these cells are important in maintaining ovarian homeostasis and they play critical roles in ovarian physiological events, such as folliculogenesis, ovulation, corpus luteum formation and regression. As females age, ovarian tissue microenvironment is typified by chronic inflammation with exacerbated ovarian fibrosis. In response to specific danger signals within aged ovaries, macrophages polarize into different M1 or M2 phenotypes, and specialize in unique functions to participate in the ovarian aging process. In this review, we will focus on the physiologic roles of MΦs in normal ovarian functions. Furthermore, we will discuss the roles of MΦs in the process of ovarian senescence, as well as the novel techniques applied in this field.

## 1 Introduction

The ovary is a key organ in female reproductive system. It produces oocytes and multiple reproductive hormones including estrogen, progesterone and androgens (1). Unlike other organs in the body, the mammalian ovary is one of the first organs to undergo early senescence. Ovarian aging is characterized by ongoing reduction in follicle number and steroid hormones generation, with deterioration of oocyte quantity and quality (2–4). In humans, ovarian function usually begins to decline around age of 35 years, and deteriorates after 37 years old, ultimately leading to endocrine dysfunction, fertility loss and menopause (1, 3, 5). It is noteworthy that in recent years, a growing body of women opt to postpone childbearing to the later stage of life partially due to social factors. As a result, the decline of female fertility due to ovarian aging represents a great challenge in reproductive medicine for which there is no reliable treatment (4, 6, 7). So far, the molecular mechanisms underpinning ovarian aging remain unclear.

Macrophages (MΦs) are a central population of leukocytes in the innate immune system, with high levels of heterogeneity and plasticity in various tissues (8–11). They are the most abundant immune cells in mammalian ovaries (8). In addition to their general functions in infection, injury and inflammation, increasing evidence has suggested critical roles of MΦs in multiple aspects of ovarian physiology, including folliculogenesis, ovulation, corpus luteum formation and regression (12, 13). Notably, along with advancing maternal age, ovarian microenvironment is characterized by chronic inflammation with exacerbated stromal fibrosis (14–17). In response to tissue-derived stimuli such as inflammatory cytokines/chemokines and Th2-type cytokines, ovarian MΦs can polarize into different M1 or M2 phenotypes, and specialize in unique functions to participate in ovarian senescence (9, 14). It has become increasingly clear that during reproductive senescence, the perturbation of M1 and M2 phenotypes is closely associated with ovarian aging (8, 9, 14). Therefore, we summarize the physiologic roles of MΦs in normal ovarian functions. Moreover, we discuss the roles of MΦs in ovarian senescence, as well as novel techniques applied in this field.

## 2 Overview of macrophages

MΦs constitute a vital component of innate immune system, and play important roles during infections and inflammation. They are often distributed in multiple tissues/organs of the body (8, 18). Previously, MΦs are thought to solely originate from monocytes, which are derived from precursors of bone marrow. Monocytes circulate in blood for several days and ultimately migrate to specific tissues where they differentiate into MΦs. However, in addition to monocyte-derived MΦs, it has recently reported that some MΦs within tissues arise from yolk sac and fetal liver during embryogenesis (9, 11). Notably, MΦs display high levels of plasticity, as reflected by that they phenotypically and functionally adapt to diverse tissue-specific environments. These local MΦ populations are essential for maintaining tissue homeostasis (11, 19). So far, due to MΦs’ complex property, the biology of MΦs is still not fully understood.

### 2.1 Plasticity, polarization and phenotype of macrophages

MΦs display strong heterogeneity and plasticity in their phenotypes and functions when exposed to various tissue microenvironments (20). In response to microorganism, microenvironmental stimuli/signals, MΦs switch from one phenotype to another, reflecting MΦs’ plasticity (11, 14, 19). Based on surface markers and biological activities, MΦs are commonly divided into two distinct subpopulations, including classically activated (M1) and alternatively activated (M2) MΦs (19, 21). Traditionally, M1 MΦs are induced by pro-inflammatory signals, such as interferon-γ, tumor necrosis factor-α (TNF-α), granulocyte-macrophage colony stimulation factor (GM-CSF), or lipopolysaccharide. In contrast, M2 MΦs are induced by anti-inflammatory signals such as IL-4, IL-13 and IL-10. Besides, IL-21 and IL-33 can also drive M2 polarization. Under the stimulation of various stimuli, M2 MΦs can be further divided into four subsets, M2a, M2b, M2c and M2d (12, 19). Specifically, M2a subset is induced by IL-4 or IL-13, whereas M2b subset is induced by immune complexes, Toll-like receptor (TLR) ligands, or IL-1 receptor agonists (IL-1Ra). M2c subset is induced by glucocorticoids, IL-10 or TGF-β. Finally, M2d subset, also known as tumor-associated macrophages, is induced by TLR ligands, A2 adenosine receptor agonists, or IL-6 (11, 12).

### 2.2 Function of macrophages

Typically, activated MΦs express a variety of receptors, including co-stimulatory and antigen presenting molecules (e.g. CD80, CD86, major histocompatibility complex I/II), chemotactic/activating cytokine receptors, pattern recognition receptors, and opsonic receptors (12). MΦs perform diverse functions during inflammation, infection and injury (22–24). Firstly, they defense against microorganisms by engulfing pathogens, and removing dying cells. Secondly, they process and present antigens to helper T-cells and stimulate them. Thirdly, they produce various cytokines, chemokines, growth factors and enzymes to recruit immune cells, as well as to facilitate vasculogenesis, tissue remodeling and repair (11, 20).

With MΦs polarization into M1 and M2 phenotypes, they exhibit enormous functional heterogeneity (11, 20). Specifically, M1 MΦs have a pro-inflammatory phenotype. They generate various chemokines and pro-inflammatory cytokines, such as TNF-α, IL-1α/β, IL-6, IL-12, IL-18 and IL-23, and possess enhanced antigen-presentation capabilities to participates in adaptive immune response (11). Additionally, M1 MΦs produce lysosomal enzymes and inducible nitric oxide synthase (iNOS) to eliminate pathogens (21). By stark contrast, M2 MΦs have an anti-inflammatory phenotype. They produce anti-inflammatory cytokines including IL-10 and transforming growth factor β (TGF-β), fibroblast growth factor (FGF) and platelet-derived growth factor (PDGF), which facilitate inflammation resolution, tissue repair and fibrosis (11, 19, 21). Moreover, diverse M2 subpopulations perform differential functions (11, 12). M2a subset suppresses inflammation and promotes tissue remodeling/repair through producing IL-10 and TGF-β (21). M2b subset simultaneously secretes pro-inflammatory and anti-inflammatory cytokines including IL-1β, IL-6, TNF-α and IL-10, which are responsible for immune regulation (25, 26). In contrast, M2c subset can phagocytose apoptotic bodies and repair injured tissues (25, 27). Also, they exert a strong anti-inflammatory effect via releasing TGF-β and IL-10 (11). M2d subset produces TGF-β, IL-10, and vascular endothelial growth factor (VEGF), which promotes tumor angiogenesis and metastasis (28).

## 3 Macrophages and ovarian function

MΦs are the most abundant immune cells in mammalian ovaries. The number and distribution of these cells change during ovarian cycles (8, 29). Accumulated evidence has revealed that MΦs are key players in various aspects of ovarian physiology (12, 13, 30). **Table 1** summarizes the roles of MΦs subsets in normal ovarian function.

**Table 1:** Roles of macrophage subsets in normal ovarian function and ovarian aging.

Macrophage subsets	Effects on normal ovarian function	Effects on ovarian aging	References
M1	Promote vascular growth, follicle development Promote luteolysis Induce primordial follicles activation	Impair oocyte quality Increase atretic follicle number Reduce growing follicle number	Ono et al. (31) Orecchioni et al. (19) Care et al. (32) Skarzynski et al. (33) Xiao et al. (34)
M2	Promote luteinization, progesterone production Maintain follicles in a dormant status	Promote ovarian ECM deposition and fibrosis Improve growing follicle number, oocyte quality, AMH and estrogen levels Reduce atretic follicle number	Ingman et al. (35) Zhang et al. (14) Xiao et al. (34) Umehara et al. (36)

ECM, extracellular matrix; AMH, anti-mullerian hormone.

### 3.1 Roles of macrophages in folliculogenesis and follicular atresia

**Figure 1:**
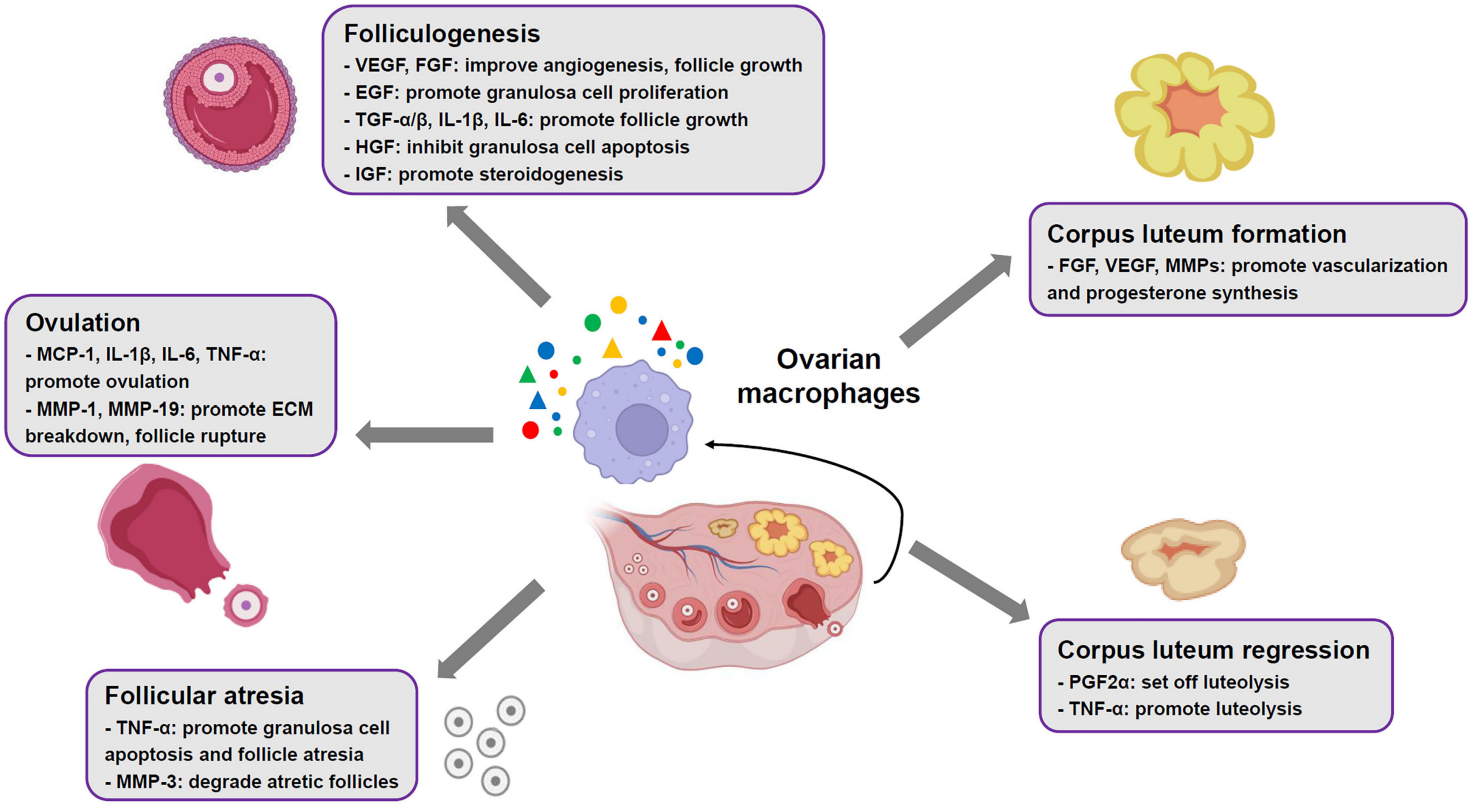
Roles of ovarian macrophages in normal ovarian functions. In mammalian ovaries, the macrophages contribute to ovarian physiological events, such as folliculogenesis, ovulation, corpus luteum formation and regression, through production of multiple cytokines and mediators.

Human and animal studies have suggested an abundant presence of MΦs in thecal layer of growing follicles (9). Ovarian MΦs contribute to follicular growth via their derived cytokines and growth factors, involving VEGF, hepatocyte growth factor (HGF), FGF, epidermal growth factor (EGF), TGF-α/β, insulin-like growth factor (IGF), IL-1β and IL-6 (**Figure 1**). These factors promote proliferation of granulosa cells, vascular growth, follicle development and production of steroid hormones, whereas inhibit apoptosis of granulosa cells in the ovary (9, 12, 37, 38). Additionally, recent studies have identified distinct MΦs subpopulations in mouse ovaries, which play essential roles in ovarian homeostasis and functions (9, 31, 39). It is revealed that in young mouse ovaries, the proportion of CD11c^+^ M1 MΦs increases significantly around developing follicles, while the proportion of CD206^+^ M2 MΦs does not. Moreover, depletion of CD11c^+^ M1 MΦs using diphtheria toxin injection in mice leads to follicular impairment, vasculature impairment and ovarian hemorrhage, whereas depletion of CD206^+^ M2 MΦs does not (31). This implies that M1 subset plays an important role in maintenance of follicles development and ovarian physiology.

In mammalian ovaries, only a fraction of primordial follicles achieve ovulation, while more than 99% of follicles undergo atresia (40). Studies have revealed that ovarian MΦs increasingly infiltrate granulosa cell layers surrounding atretic follicles (9, 41). This migration/recruitment event is mediated by IL-33, which is mostly generated by endothelial cells adjacent to atretic follicles (42, 43). The infiltrated MΦs facilitate granulosa cell apoptosis and follicle atresia by secretion of TNF-α (44) (**Figure 1**). Subsequently, these cells are responsible for removing apoptotic cells and degrading atretic follicles through production of matrix metalloproteinases (MMPs) like MMP-3[9] (**Figure 1**).

### 3.2 Roles of macrophages in ovulation, corpus luteum formation and regression

A surge of luteinizing hormone (LH) secreted by pituitary gland initiates ovulation, which involves preovulatory follicles rupture at the apex and extrude cumulus cell-oocyte complex (45). Animal studies have revealed that ovarian MΦs actively participate in ovulation (12), as colony stimulation factor-1 knockout female mice with elimination of MΦs demonstrate compromised ovulation (46). During ovulatory process, LH surge facilitates production of multiple chemoattractants by granulosa cells, such as chemokine (C-X-C motif) ligand 10, C-C-motif ligand-20, monocyte chemoattractant protein-1 (MCP-1), IL-1 and IL-6. As a result, a large number of ovarian MΦs are recruited to preovulatory follicles by the chemoattractants (34, 47). These cells promote ovulation through secreting MCP-1 and pro-inflammatory cytokines like IL-1β, IL-6 and TNF-α, which simultaneously amplify recruitment effects (**Figure 1**). Besides, recruited MΦs produce MMPs like MMP-1 and MMP-19 contributing to extracellular matrix (ECM) breakdown, which facilitates follicle rupture and oocyte extrusion (9, 45) (**Figure 1**).

Following ovulation, the remains of ovarian follicles undergo tissue remodeling, involving luteinization of follicular theca cells and granulosa cells, and vascularization, to develop a corpus luteum (CL) (48). The CL functions as a temporary endocrine structure through generation of high levels of progesterone and moderate levels of estradiol and inhibin A (9, 48). Evidence from human and animal models has suggested a close relationship between ovarian MΦs and CL development and function (9, 32, 49, 50). Upon stimulation of chemokine MCP-1 and C-C-motif ligand-2 (CCL-2), and GM-CSF, MΦs accumulate and exhibit an activation status in theca-lutein layer of CL (49, 50). They support vascularization of luteal cells and synthesis of progesterone through releasing of FGF, VEGF and MMPs (9, 51) (**Figure 1**). It is worth noting that disruption of M2 phenotype polarization in TGF-β-deficient female mice leads to impaired luteinization and reduced progesterone production in CL, implying that M2 subset is a key player in developing CL (9, 35). If the oocyte is not fertilized, the CL subsequently undergoes degeneration. This process is also called luteolysis, which is set off by prostaglandin F2α (PGF2α) (32). Ovarian MΦs are found to polarize towards M1 phenotype that facilitate PGF2α production through secretion of TNF-α, thereby promoting luteolysis, indicative of importance of M1 subset in CL regression (19, 32, 33) (**Figure 1**). Conversely, if the oocyte is fertilized and implantation occurs, human chorionic gonadotropin produced by syncytiotrophoblast prevents MΦs accumulation, resulting in the CL maintenance (12).

## 4 Macrophages and ovarian aging

As a key reproductive organ in females, the ovary, however, ages early in life (2, 3, 39). Until now, the mechanisms underlying ovarian aging have not been fully elucidated. The studies of ovarian MΦs in both mice and humans are constrained, as their number is very small and they display high heterogeneities in phenotypes (9, 34). Recently, the advance of high-throughput sequencing techniques has made it possible to investigate ovarian MΦs at the single-cell level. Using these novel technologies, emerging studies highlight critical roles of MΦs in ovarian aging (9, 39). **Table 1** summarizes the roles of MΦs subsets in ovarian aging process.

### 4.1 Macrophages dictates the inflammatory milieu within the aging ovary

**Figure 2:**
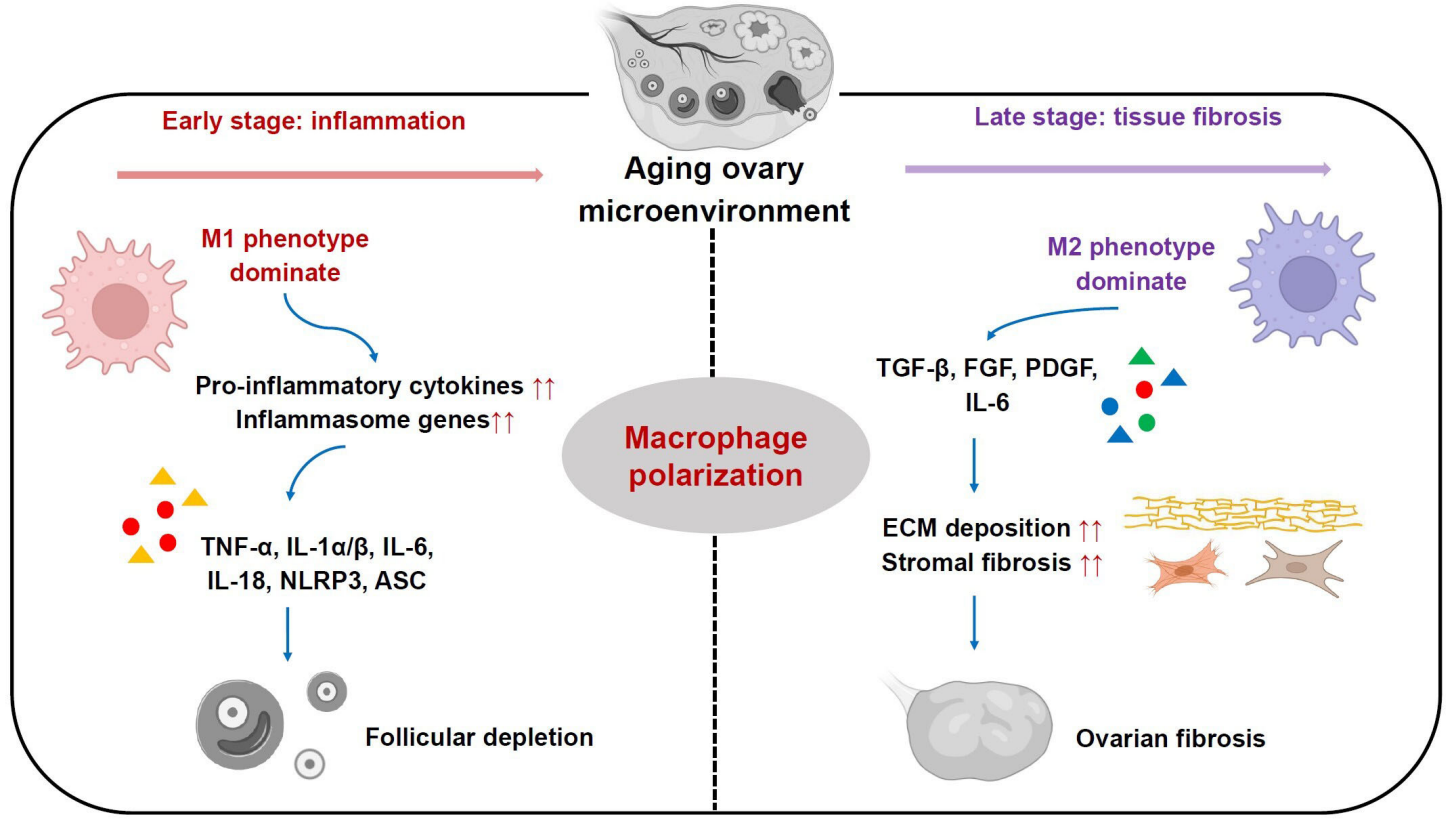
Roles of ovarian macrophages and their subsets in ovarian aging process. In the early stage, M1 phenotype subset is dominant and plays a pro-inflammatory role by secreting pro-inflammatory cytokines, including TNF-α, IL-1α/β, IL-6, IL-18, which in turn boost elevated expression of inflammasome genes like NLRP3 and ASC. In the late stage, M2 phenotype subset is more predominant and participates in ECM deposition and stromal fibrosis, ultimately leading to ovarian fibrosis.

Mounting evidence suggests that ovarian aging in mammals is associated with a sterile chronic inflammation in ovaries, which adversely affects ovarian function and oocyte quality (14, 16, 17, 52, 53). Recent studies have revealed that as female C57BL/6 mice age (from two to eighteen months old), levels of pro-inflammatory cytokines, including TNF-α, IL-1α/β and IL-6, were significantly elevated in serum and ovary (53). Furthermore, similar alterations were seen in levels of inflammasome genes, involving nucleotide-binding domain and leucine rich repeat containing family, pyrin domain containing 3 (NLRP3) and apoptosis-associated speck-like protein containing a CARD (ASC). They are capable of boosting production of pro-inflammatory cytokines IL-1β and IL-18 (53). Notably, the increased levels of these pro-inflammatory cytokines and inflammasome genes are found to be closely related to declined follicle reserve along with reproductive senescence (53, 54) (**Figure 2**). Nonetheless, the mechanisms underlying persistent inflammatory condition in aged ovaries remain elusive.

Recently, it has been proposed that MΦs are responsible for age-associated inflammation within the ovary (9, 14, 39). In comparison with reproductively young mice (2-month-old), there is a conspicuous increase in the MΦs proportion within ovaries from reproductively aged mice (12-month-old), which were driven by CCL-2 and chemokine ligand-5 (16, 53). These cells showed an activation status reflected by secreting high levels of pro-inflammatory cytokines including IL-1, IL-6 and TNF-α, exacerbating granulosa cell apoptosis and follicular depletion (9, 53) (**Figure 2**). In addition, other mouse studies demonstrated the presence of a hyperactivated form of MΦs, multinucleated giant cells, in ovarian stroma over the course of reproductive ageing (15, 16). However, the mechanisms underlying activation status of ovarian MΦs as females age remain poorly understood. Several studies in mouse models have revealed that excessive accumulation of incompletely digested cell debris like lipofuscin, and low molecular weight hyaluronan fragments from ECM, might be the drivers of intensive activation status of MΦs during ovarian aging (54–56).

### 4.2 Macrophages contribute to ovarian fibrosis during reproductive aging

In addition to chronic inflammation, stromal fibrosis within ovaries is another hallmark of mammalian ovarian senescence (16, 57). Ovarian stroma is broadly considered as non-follicular components of the ovary, including immune cells, blood vessels, nerves, and ovary-specific components like spindle-shaped stromal cells, stem cells and ECM (39, 57–59). It serves as an essential supporting tissue for maintaining ovarian homeostasis and functions (9). Previous works in mice and humans have shown an increase in stromal fibrosis and an excessive deposition of ECM components in reproductively old ovaries, which are associated with reduced follicle number, impaired follicle development and ovarian dysfunction (16, 60). Besides aging-related increase of fibrosis, a marked increase in monocyte recruitment and a shift in MΦs phenotype towards M2 were found within ovaries from reproductively aged mice (18 months) relative to young mice (3 months). Subsequently, the monocyte-derived MΦs and M2 subpopulation become more predominant with reproductive aging. They promote ovarian ECM deposition and fibrosis by secreting high levels of TGF-β, FGF, PDGF as well as pro-inflammatory cytokines like IL-6 (9, 14) (**Figure 2**). Moreover, recent transcriptomics data from ovarian aging models of cynomolgus monkey has further confirmed these findings (61).

### 4.3 Macrophage polarization is associated with ovarian aging

Recent animal studies have revealed that shifted MΦs subpopulations with different phenotypes resulting from their polarization play critical roles in ovarian senescence (17, 62, 63) (**Figure 2**). It has been found that in ovaries of young female ICR mice at 8 weeks old, M1 phenotype mainly functions in primordial follicles activation, while M2 phenotype functions in maintenance of follicles in a dormant status (34). They perform differential functions through MΦs-derived distinct extracellular vesicles (EVs) (34). Notably, with female ICR mice aged at 10 months old, the percentage of M1 phenotype within ovaries was increased relative to young females, which is accompanied by an elevated expression of several pro-inflammatory genes including *IL-6*, *TNF-α*, *IL-17*, *iNOS*, *ASC* and *NLRP3*. By contrast, M2 phenotype did not show significant changes (34). Furthermore, the addition of M2-derived EVs into these old mice could enhance M2 phenotype proportion, which ultimately rescued growing follicle number, oocyte quality, serum anti-mullerian hormone and estrogen levels. Meanwhile, it could reduce atretic follicle number, and levels of pro-inflammatory genes expression involving *IL-1β*, *IL-6*, *iNOS* and *TNF-α*. This implies that the perturbed dynamics of M1 and M2 subpopulations are actively involved in ovarian functional decay with reproductive senescence (34) (**Figure 2**). However, another recent study showed inconsistent results using C57BL mouse models with advanced reproductive age at 12-16 months old. This work revealed high expression levels of inflammatory chemokines (CCL-2, CCL-3, and CXCL-2), pro-inflammatory cytokines TNF-α and IL-6, and Th2-type cytokines IL-4 and IL-13 in the aged ovarian stroma, which drove MΦs polarization. As a result, there were increase numbers of both M1 and M2 subpopulations, while M2 number was more predominant within ovarian stroma of old mice. These cells promoted ovarian fibrosis by stimulating fibrotic collagen deposition (**Figure 2**). Following suppression of the M2 subpopulation by antifibrosis drug (BGP-15), it was observed that ovarian fibrosis was reversed, and ovarian function and female fertility were finally improved (36). The discrepancy of these findings may be due to dynamic changes of M1/M2 phenotype in different stages of ovarian aging. In early phase, M1 phenotype is dominant and plays a pro-inflammatory role, whereas in late phase, M2 phenotype is more predominant and participates in inflammation resolution, tissue remodeling and repair in aging ovaries (9, 14, 64) (**Figure 2**). Thus, regulation of the balance of MΦs polarization may be a potential therapeutic strategy for reproductively aged women to restore ovarian function and fertility.

Until now, most studies on ovarian MΦs during aging are focused on animals, while human studies are still scarce, probably due to difficulties in obtaining human samples (17, 65). A recent human study has shown that in comparison to premenopausal women (30-50 years old), MΦs number was remarkably higher in ovarian stroma in women at early (55-59 years old) and late menopausal (60-85 years old) stages. These MΦs produce high levels of interleukin 16 (IL-16), a pro-inflammatory and chemotactic cytokine, indicative of an inflammatory role of ovarian MΦs during female aging (66). Therefore, more investigations in human models are still required to elaborate the roles of different MΦs phenotypes during ovarian aging.

Remarkably, recent studies have successfully applied single-cell RNA sequencing (ScRNA-seq) technology to transcriptomic analysis of ovaries, follicles as well as MΦs subpopulations (61, 67). Diverse methods have been further derived from ScRNA-seq, involving massively parallel single-cell RNA sequencing (MARS-seq), CEL-seq, Drop-seq, and Slide-seq (68–71). Additionally, *in vivo* imaging techniques, like intravital two-photon imaging and multichannel spinning-disk confocal intravital microscopy, will allow researchers to track ovarian MΦs subpopulations and investigate their polarization behaviors in real time within aging ovaries (72–74). Hence, these advanced technologies may assist an in-depth understanding of the roles of different MΦs subsets in ovarian senescence.

## 5 Conclusion and perspectives

Ovarian MΦs play pivotal roles in normal ovarian functions and ovarian aging. During reproductive senescence, danger signals within aged ovaries induce MΦs polarization into different M1/M2 phenotypes. Perturbation of balance of M1/M2 phenotypes in aged ovaries dictates chronic inflammatory milieu concurrent with stromal fibrosis, leading to follicular loss and ovarian dysfunction. To regulate the balance between M1 and M2 subsets might be a promising therapeutic strategy for women with advanced reproductive age. Future studies are still needed to further unravel the roles of MΦs in ovarian aging and develop a new approach to ameliorate ovarian decay.

## Author contributions

MT: Writing – original draft. MZ: Writing – review & editing. YS: Supervision, Writing – review & editing.
